# A New Combination for the Treatment of Hepatic Derangements

**Published:** 1908-12

**Authors:** Arthur Wegefarth

**Affiliations:** Associate Professor of Clinical Medicine in Baltimore Medical College. Attending Physician Maryland General Hospital, Baltimore


### ABSTRACT.

### A New Combination for the Treatment of Hepatic Derangements.

By ARTHUR WEGEFARTH, M.D.

Associate Professor of Clinical Medicine in Baltimore Medical College.
Attending Physician Maryland General Hospital, Baltimore.

NEARLY a year ago I began the clinical investigation in the therapeutic value of a mixture of acid sodium oleate, sodium salicylate, phenolphthalein, and menthol, for the relief of derangements of the hepatic mechanism, which, in my experience, have been somewhat difficult to satisfactorily treat by purely medicinal means. The formula used in my clinical experiments was as follows:

| | | |
|---|---|---|
| Acid sodium oleate, .......... | 1½ | grains |
| Sodium salicylate ............. | 1½ | grains |
| Phenolphthalein ............... | 1-3 | grains |
| Menthol ....................... | 1-10 | grains |

combined in a soft mass and exhibited as a pill with a thin coating. This combination has since been offered upon the market under the name of "Pill Cholelith." The following three cases fairly illustrate the conditions in which I have found this combination very successful:

CASE 1.—Mary E. C., married, age 44 years, came to me complaining of being constantly sick at the stomach, very nervous, suffering from headache. She admitted being constipated, the bowels moving only every two or three days. She stated that this condition had been present practically all of the time for the last six years. Her tongue was heavily coated, her face thoroughly hyperemic; there were soft acne pustules on the side of her nose. And she had lost a great deal of weight in the past twelve months. An examination showed that the heart was normal; the urine analysis was negative; her liver was noticeably diminished in size and hard and irregular in outline. I considered this a case of cirrhosis, and a little diplomatic questioning disclosed the fact that the patient was addicted to drink. I started the treatment with administration of one of the pills referred to above every three hours. In less than a week the patient's condition was greatly improved. Constipation was relieved, bowel movement becoming quite frequent; the patient's appetite was much better and sleeplessness of which she complained was much relieved. At this time, the patient disappeared and, I learned later had started to drink heavily. Two months afterwards, I was called to see the patient again and found her original condition reëstablished. Tongue was again heavily coated; she was constipated; the nervousness was once more present. The pills were again administered one every three hours, and the patient improved rapidly. I am convinced that if this patient could have been controlled and subjected to a continued course of treatment, that she would have completely recovered.

CASE 2.—Henry F. W., age 35. Complained of constant headache in the occipital region; chilly feeling; aching sensation over the body; constipation constantly present. From his description it is believed that he had periodic attacks of jaundice; he had no appetite; his tongue was heavily coated; the conjunctiva of each eye was decidedly yellow, showing jaundice. This was evidently a case of hepatic congestion, and the pills were ordered one every three hours. Shortly after the treatment was instituted the patient's condition rapidly improved, which good reports were continued as long as the treatment was kept up. The case could not be followed as he was exceedingly irregular in his visits. As a consequence this case is not very satisfactory, but illustrates the good effect of the combination in such conditions.

CASE 3.—Mrs. P. H. F., age 54, married. Gave a history of having gallstone colic and jaundice. Had just recovered from a severe attack of colic accompanied by pain and jaundice. Her tongue was very heavily coated; she was constantly nauseated and badly constipated; the region over the liver was very tender. The pills were administered one every three hours, and in a very short time the tongue cleared, the bowel movement became normal, nausea disappeared, the appetite returned and the stain on the tissues from the bile cleared up very nicely.

This case illustrates very well the satisfactory results from the combination in cases of gallstone colic.

It has been my habit to recommend the pill every three hours, taken with large amounts of hot water, to prohibit all alcoholic drinks and all indigestible foods. The beneficial effect of the combination upon the liver and intestines is quite marked.

SOUTHEAST CORNER EUTAW PLACE AND NORTH AVE.

